# Composite neuroendocrine tumor and adenocarcinoma of the rectum

**DOI:** 10.1186/s13000-017-0674-8

**Published:** 2017-12-11

**Authors:** Hiromi Kanno-Okada, Tomoko Mitsuhashi, Katsuhiro Mabe, Tadakazu Shimoda, Yoshihiro Matsuno

**Affiliations:** 1 Department of Surgical Pathology, Hokkaido University Hospital, North 14, West 5, Kita-ku, Sapporo, Hokkaido 060-8648 Japan; 2 Department of Gastroenterology, Hakodate National Hospital, Kawahara-cho 18-16, Hakodate, Hokkaido 041-8512 Japan; 3 Division of Pathology, Shizuoka Cancer Center, Shimonakakubo 1007, Izumi-cho, Suntou-gun, Pref., Shizuoka, 411-8777 Japan

**Keywords:** Neuroendocrine tumor, Adenocarcinoma, Rectum

## Abstract

**Background:**

Although adenocarcinomas showing neuroendocrine differentiation or those mixed with high-grade neuroendocrine carcinoma (NEC) are sometimes encountered, composite tumors comprising neuroendocrine tumor (NET) Grade 1 and adenocarcinoma are exceedingly rare.

**Case presentation:**

A 64-year-old male presented after testing positive for fecal occult blood at a medical check-up. A biopsy demonstrated the presence of a NET and endoscopic submucosal dissection was undertaken. Histologic examination revealed that a well differentiated tubular adenocarcinoma was present in addition to the NET. Furthermore, histological transition between the two tumors was evident. Accordingly, this case was considered to be a composite tumor comprising NET and adenocarcinoma.

**Conclusion:**

Composite tumors consisting of NET Grade 1 and adenocarcinoma are exceedingly rare, and only a few examples have been reported hitherto.

## Background

Gastrointestinal neuroendocrine tumor (NET) is a well differentiated neuroendocrine neoplasm commonly occurring in the rectum and appendix. In the latest World Health Organization (WHO) Classification, NET Grade 1 (G1) is defined as low-grade malignancy [1]. Although adenocarcinomas showing neuroendocrine differentiation or those mixed with high-grade neuroendocrine carcinoma (NEC) are sometimes encountered, composite tumors comprising NET G1 and adenocarcinoma are exceedingly rare. To the best of our knowledge, only 10 cases of possible composite NET and adenocarcinoma arising in the colorectal region have been reported in the English literature [2–6].

Here we report a case of composite NET G1 and adenocarcinoma of the rectum in which the two components showed histological transition.

## Case presentation

### Clinical history

**Fig. 1:**
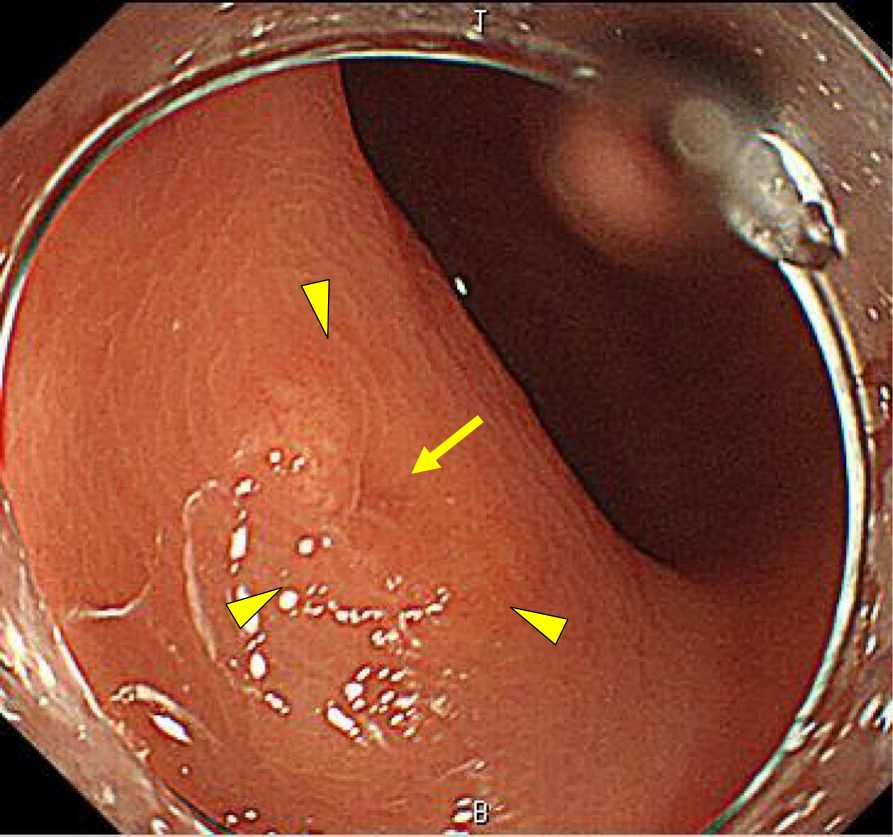
Endoscopic view of the yellowish submucosal tumor (arrow heads) with a slight central depression (arrow)

A 64-year-old male with a history of benign prostatic nodular hyperplasia presented after testing positive for fecal occult blood at a medical check-up. Colonoscopy performed at the previous hospital had demonstrated a yellowish submucosal tumor in the lower rectum. Biopsy of the tumor revealed a typical NET, and the patient was referred to our hospital. Endoscopic examination showed a 9-mm yellowish submucosal tumor with a slight central depression (Fig. 1) and unclear demarcation between the lesion and the normal mucosa. Endoscopic submucosal dissection (ESD) was performed after marking around the lesion, and *en bloc* resection was achieved without any adverse events. After ESD, PET-CT and ultrasonography showed no obvious metastatic lesion in the lymph nodes and other organs. The patient is currently doing well without any recurrence or metastasis at 18 months after ESD.

### Pathologic findings

Histologically, the endoscopic biopsy specimen of the rectal mucosa taken at the previous hospital showed well differentiated NET with an insular or trabecular growth pattern (data not shown). The tumor cells were cuboidal (or polygonal), with uniform round nuclei and eosinophilic granular cytoplasm. Mitotic figures were sparse, and no necrosis was evident. Immunohistochemically, the tumor cells were positive for CD56 and synaptophysin, and negative for chromogranin A. The Ki-67 labeling index was approximately 2.0%. Accordingly, the lesion was diagnosed as NET G1. The specimen showed no atypical glandular epithelium or adenocarcinoma.

**Fig. 2:**
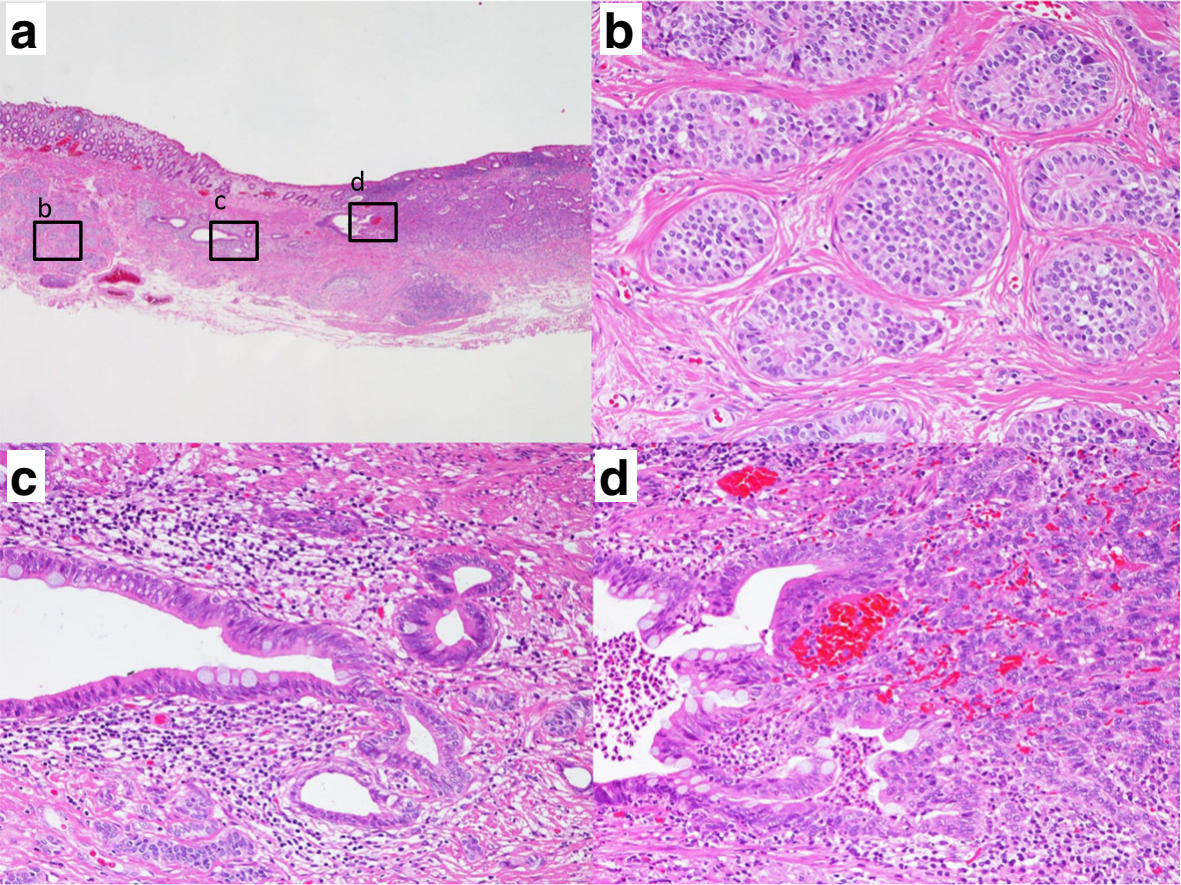
Representative micrographs of the submucosal lesion. (**a**) Low-power view shows a typical neuroendocrine tumor (**b**), an atypical glandular component (**c**) and an admixture of the two components (**d**). The surface epithelium appears intact. *b* High-power view of the typical neuroendocrine tumor in the submucosal layer. This component is composed of uniform tumor cells with “salt-and-pepper” rounded nuclei and eosinophilic granular cytoplasm. *c* High-power view of the atypical glandular component in the submucosal layer. This component shows atypical epithelial cells with enlarged nuclei and coarse chromatin, forming irregular glands. At the periphery, trabeculae of endocrine cells are also evident. *d* Atypical glandular components show histologic transition to the typical neuroendocrine tumor component with a rosette-like or reticular growth pattern

Macroscopic observation of the ESD specimen showed a yellowish submucosal tumor measuring 9 × 6 mm with a slight central depression. Histologically, the lesion was heterogeneous, comprising two different components (Fig. 2a). One component was NET with an insular or trabecular growth pattern, being located in the mucosa and submucosa. The NET cells had uniform “salt-and-pepper” rounded nuclei and eosinophilic granular cytoplasm (Fig. 2b), similar to the biopsy specimen. Mitoses were infrequent (0 /10HPFs) and there was no necrosis. The other component was glandular in nature, comprising abnormal epithelial cells with enlarged nuclei, coarse chromatin and several mitoses (2/ 10HPFs)forming irregular glands (Fig. 2c). This glandular component was present in the submucosa and muscularis mucosa, and showed histologic transition to the NET component with a rosette-like or reticular growth pattern (Fig. 2d). This intermediate component didn’t show mitosis (0/ 5HPFs) or necrosis. Much of the surface epithelium was atrophic, but mostly intact.

**Fig. 3:**
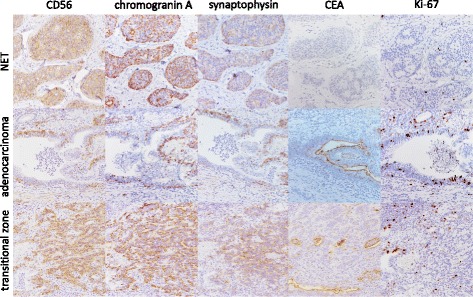
Immunohistochemical staining of the lesion. (Top) The neuroendocrine tumor component shows positivity for CD56, chromogranin A and synaptophysin, and is negative for carcinoembryonic antigen (CEA). The Ki-67 labeling index was low. (Middle) The adenocarcinoma component shows partial positivity for CD56, chromogranin A and synaptophysin at the periphery and positivity for CEA. The Ki-67 labeling index was 37.0%. (Bottom) The transitional zone shows positivity for CD56, chromogranin A and synaptophysin, and partial positivity for CEA. The Ki-67 labeling index was 1.6%

Immunohistochemically, the NET component was positive for CD56, chromogranin A and synaptophysin, and negative for carcinoembryonic antigen (CEA) (Fig. 3, top) and CDX-2 (data not shown). The Ki-67 labeling index was 0.5% (Fig. 3, top), corresponding to NET G1.

On the other hand, the adenocarcinoma component was immunopositive for CEA (Fig. 3, middle) and CDX-2 (data not shown). CD56, chromogranin A and synaptophysin were partially positive at the periphery of the glandular component (Fig. 3, middle). The Ki-67 labeling index was 37.0% (Fig. 3, middle). Accordingly, the latter component was confirmed to be a well differentiated tubular adenocarcinoma, not normal glands or tubular adenoma. The transitional zone was positive for CD56, chromogranin A and synaptophysin, partially positive for CEA (Fig. 3, bottom) and negative for CDX-2 (data not shown). The Ki-67 labeling index was 1.6% (Fig. 3, bottom). We concluded that this case was a composite tumor comprising NET G1 and adenocarcinoma. The transitional component didn’t fulfill the criteria of NEC. Adenocarcinoma had invaded to the superficial submucosal layer (less than 1000 μm from the muscularis mucosa) at its deepest point. There was no identifiable vascular or lymphatic invasion. The resection margin was negative for both of the tumor components.

## Discussion

NET is a neuroendocrine neoplasm with low-grade malignancy, being common in the rectum and appendix [7]. Histologically, the tumor cells have uniform rounded nuclei and granular cytoplasm, and show a solid, insular, acinar or trabecular growth pattern. Sometimes, they show rosette-like and/or glandular growth patterns and a rich vascular stroma. In the WHO Classification, NET G1, NET G2 and NEC are all classified as neuroendocrine neoplasms. However, NET and NEC have been considered to be different entities because of differences in histogenesis, malignant potential and gene alterations [7]. Although association of NEC with adenoma or adenocarcinoma is not uncommon, composite tumors comprising NET G1 and adenocarcinoma are exceedingly rare. In the present case, NET G1 and a tubular adenocarcinoma were intermixed without a distinct boundary, and showed histologic transition between them. Accordingly, we concluded that this case was a composite tumor of NET G1 and adenocarcinoma. This case appeared to be different from “adenocarcinoid” or “goblet cell carcinoid”, most of which arise in the appendix. Adenocarcinoid exhibits histological features of both adenocarcinoma and NET, but is composed of mucin-containing goblet-shaped cells or signet-ring-like cells [8–10].

To our knowledge, about 10 cases of composite NET and adenocarcinoma arising in the colorectal region have been reported [2–6]. In addition to these colorectal cases, composite tumors of the stomach, small intestine, anal canal and gallbladder have also been documented [4, 11, 12]. In some cases, however, it was not clear if the two components were intermixed, and therefore the possibility of collision tumor of NET and adenocarcinoma could not be excluded. Furthermore, some reports did not give information about the grade of NET, including details of the mitotic count and the Ki-67 labeling index, and thus the possibility of mixed adenoneuroendocrine carcinoma (MANEC) could not be ruled out. According to the WHO Classification (2010), MANECs have both gland-forming epithelial and neuroendocrine components, with one component exceeding 30%, and both of components are defined as carcinoma with high-grade malignancy [1]. The present case was not a MANEC because the NET component was apparently a well differentiated NET G1 with a low mitotic index and a low Ki-67 labeling index. In addition, composite tumors comprising NET and adenocarcinoma should be distinguished from adenocarcinoma with focal neuroendocrine differentiation. Differences in clinical outcome can be expected between the present type of tumor, MANEC, and adenocarcinoma with focal neuroendocrine differentiation.

Another differential diagnosis that needs to be considered is glandular differentiation of NET. NET is known to show glandular differentiation or mucin production [13]. In the present case, the glandular component showed apparent nuclear atypia, discrete gland formation, and positivity for CEA and CDX-2 without a “salt-and-pepper” chromatin pattern. In addition, the Ki-67 labeling index was apparently higher than that of a typical NET. Accordingly, we were able to exclude the possibility of glandular differentiation of NET. Furthermore, the possibility that the atypical glandular component could be an epithelial cell inclusion or ectopic glands, rather than adenocarcinoma, was ruled out because this component showed moderate atypia and a much higher Ki-67 labeling index than normal epithelial cells.

The histogenesis of composite NET and adenocarcinoma has not been fully elucidated. At least three possible hypotheses can be suggested: (a) Partial differentiation of adenocarcinoma into NET, (b) partial transformation of NET into adenocarcinoma, and (c) bidirectional transformation of common putative precursor cells into both endocrine cells and glandular epithelial cells. In the present case, neither of the specimens obtained by biopsy and ESD showed atypia in the surface epithelium, being incompatible with hypothesis (c). If the adenocarcinoma had partially differentiated into NET, then for former would have been observed in the surface epithelium. In addition, it has been reported that endocrine cells of the digestive tract are derived from local multipotent gastrointestinal stem cells, rather than migrating from the neural crest as reported previously [14, 15]. Taken together, the findings suggest that this composite tumor might have differentiated from common putative precursor cells bidirectionally at an early stage of tumorigenesis.

The prognosis of composite NET and adenocarcinoma seems to be determined by the carcinoma component, although the number of reported cases is limited [4]. In the present case, the adenocarcinoma had invaded to the superficial submucosal layer at its deepest point, but there was no vascular or lymphatic invasion. Eighteen months after ESD without additional therapy, the patient is doing well without any recurrence or metastasis.

## Conclusion

We have thus described a rectal composite tumor comprising NET and adenocarcinoma. Although only a few reports of composite tumor have been published so far, careful pathological examination may reveal more similar cases.

## Abbreviations

CEA: carcinoembryonic antigen
ESD: Endoscopic submucosal dissection
G1: Grade 1
MANEC: mixed adenoneuroendocrine carcinoma
NEC: neuroendocrine carcinoma
NET: neuroendocrine tumor
WHO: World Health Organization

## References

[1] Bosman FT, Carneiro F, Hruban RH, THeise ND, eds. WHO Classification of Tumours of the Digestive System, 4^th^ Edition. IARC Press, Lyon, 2010.

[2] Bates HR, Belter LF (1967). Composite carcinoid tumor (argentaffinoma-adenocarcinoma) of the colon: report of 2 cases. Dis Colon rectum.

[3] Klappenbach RS, Kurman RJ, Sinclair CF, James LP (1985). Composite carcinoma-carcinoid tumors of the gastrointestinal tract. A morphologic, histochemical, and immunocytochemical study. Am J Clin Pathol.

[4] Levendoglu H, Cox CA, Nadimpalli V (1990). Composite (adenocarcinoid) tumors of the gastrointestinal tract. Dig Dis Sci.

[5] Chaturvedi KU, Kaur H (1994). Composite carcinoid carcinoma of the colon--a case report. Indian J Cancer.

[6] Bhattacharjee PK, Halder S (2013). Combined adenocarcinoma-carcinoid tumor of transverse colon. J Cancer Res Ther.

[7] Vortmeyer AO, Lubensky IA, Merino MJ, Wang CY, Pham T, Furth EE, Zhuang Z (1997). Concordance of genetic alterations in poorly differentiated colorectal neuroendocrine carcinomas and associated adenocarcinomas. J Natl Cancer Inst.

[8] Toumpanakis C, Standish RA, Baishnab E, Winslet MC, Caplin ME (2007). Goblet cell carcinoid tumors (adenocarcinoid) of the appendix. Dis Colon rectum.

[9] Tang LH, Shia J, Soslow RA, Dhall D, Wong WD, O'Reilly E, Qin J, Paty P, Weiser MR, Guillem J, Temple L, Sobin LH, Klimstra DS (2008). Pathologic classification and clinical behavior of the spectrum of goblet cell carcinoid tumors of the appendix. Am J Surg Pathol.

[10] Wakahara T, Yamamoto S, Fujita S, Akasu T, Onouchi S, Moriya YA (2010). Case of advanced rectal adenocarcinoid tumor with long-term survival. Jpn J Clin Oncol.

[11] Wada A, Ishiguro S, Tateishi R, Ishikawa O, Matsui Y (1983). Carcinoid tumor of the gallbladder associated with adenocarcinoma. Cancer.

[12] Anagnostopoulos GK, Arvanitidis D, Sakorafas G, Pavlakis G, Kolilekas L, Arkoumani E, Stefanou D (2004). Combined carcinoid-adenocarcinoma tumour of the anal canal. Scand J Gastroenterol.

[13] Arai T, Kino I (1994). Histochemical and ultrastructural analyses of glandular differentiation in typical carcinoid tumor of the hindgut. Pathol Int.

[14] Modlin IM, Oberg K, Chung DC, Jensen RT, de Herder WW, Thakker RV, Caplin M, Delle Fave G, Kaltsas GA, Krenning EP, Moss SF, Nilsson O, Rindi G, Salazar R, Ruszniewski P, Sundin A (2008). Gastroenteropancreatic neuroendocrine tumours. Lancet Oncol.

[15] Brittan M, Wright NA (2002). Gastrointestinal stem cells. J Pathol.

